# A Novel Self-Binding Composite Separator Based on Poly(tetrafluoroethylene) Coating for Li-Ion Batteries

**DOI:** 10.3390/polym10121409

**Published:** 2018-12-19

**Authors:** Kaiyue Zhang, Wei Xiao, Jianguo Liu, Chuanwei Yan

**Affiliations:** 1 Institute of Metal Research, Chinese Academy of Sciences, Shenyang 110016, China; kyzhang@imr.ac.cn (K.Z.); jgliu@imr.ac.cn (J.L.); cwyan@imr.ac.cn (C.Y.); 2 Materials Science and Engineering, University of Science and Technology of China, Shenyang 110016, China

**Keywords:** self-binding coating, poly(tetrafluoroethylene), poly(ethylene) separator, cell performance, Li-ion battery

## Abstract

In this study, a novel composite separator based on polytetrafluoroethylene (PTFE) coating layers and a commercial polyethylene (PE) separator is developed for high performance Li-ion batteries. This composite separator is prepared by immersing a PE separator directly into a commercial PTFE suspension to obtain a self-binding PTFE/PE/PTFE tri-layered structure. Then, the as-prepared composite separator is further treated with a H_2_O_2_/H_2_SO_4_ solution to enhance its electrolyte affinity. The results show that the coating layer, consisting of close-packed PTFE particles, possesses a highly ordered nano-porous structure and an excellent electrolyte wettability property, which significantly enhance the ionic conductivity of the composite separator. Due to the presence of the PTFE-based coating layer, the composite separator exhibits better thermal stability compared with the PE separator, reaching the thermal-resistant grade of commercial ceramic-coated separators. By using different separators, CR2032-type unit half-cells composed of a Li anode and a LiFePO_4_ cathode were assembled, and their C-rate and cycling performances were evaluated. The cell assembled with the composite separator was proven to have better C-rate capability and cycling capacity retention than the cell with the polyethylene separator. It is expected that the composite separator can be a potential candidate as a coating-type separator for high-performance rechargeable Li-ion batteries.

## 1. Introduction

Li-ion batteries have been widely used in power-source fields, such as electronic devices, power tools, and electric vehicles [1]. To meet the growing demand for high-rate and high-power batteries, many kinds of high-performance cathode and anode materials have been produced recently [2,3]. Separators are highly important for the performance of electronic products, such as Li-ion batteries, supercapacitors, and so on [4,5,6]. However, separators, critical components of Li-ion batteries, are still being made of polyethylene (PE) or polypropylene (PP) with poor thermal stability and wettability, which raises serious concerns regarding the safety of Li-ion batteries in cases of unusual heat generation [7].

In order to solve these severe shortcomings, our team has demonstrated a novel approach using nonwoven inorganic composite separators for Li-ion batteries [8,9]. Additionally, other new separators have been explored, such as inorganic separators [10,11], polymer nano-fiber separators [12,13], and so on. Despite their good thermal stability and wettability, these new separators are still not used currently in large-scale commercial applications. Meanwhile, surface modification, particularly for surface coating, has been demonstrated commercially to be a handy and feasible method for improving the performance of polyolefin separators. In general, in the industry, ceramic particles have been adopted to coat at least one side of the conventional polyolefin separators along with fluorinated binders. Moreover, in academic research, Al_2_O_3_ [14,15,16] and SiO_2_ [17,18], as the most common ceramic materials, have been extensively studied to suppress the thermal shrinkage and mechanical breakdown of polyolefin separators. In this way, binders are commonly used to aggregate ceramic particles and immobilize them on the surfaces of polyolefin separators. In spite of this, one of the main questions researchers face is whether the presence of the binders in the coating layer reduces the porosity and blocks the Li-ion migration channels [19]. When the concentration of binders is reduced, the coating layer is easily detached from the body of the separator during charge-discharge. In addition, ceramic-coated separators have strong hygroscopicity with a higher risk of cell internal short circuit, resulting in increased difficulty of treatment during the battery pack assembling process. Therefore, an alternative coating layer with good comprehensive performance needs further development.

Polytetrafluoroethylene (PTFE) suspension, as a common binder, is widely used for electrode preparation in Li-ion batteries because of its good adhesiveness and chemical stability in organic electrolytes. In suspension, PTFE resin in the form of suspended nanoparticles plays the role of the adhesive, holding active materials together, which is different from other binders, such as polyvinylidene fluoride (PVDF) and polyvinyl alcohol (PVA). Moreover, PTFE has been extensively used in the electronics, chemical, and medical industries because of its excellent thermal stability, chemical inertness, and biological compatibility [20]. Its excellent performance and desirable attributes have attracted the attention of scholars studying separators. In recent years, an electrospun PTFE nano-fiber separator was successfully prepared as the separator for Li-ion batteries, utilizing its natural heat resistance to improve thermal stability [21], which is not straightforward in industry production. Up to now, an approach that utilizes PTFE particles to build a thermal-resistant coating layer on commercial polyolefin separators has rarely been reported but would be extremely useful.

In the present study, a novel composite separator was prepared by coating PTFE particles on both sides of a microporous PE separator without any other binders. Then, the composite separator was treated in a H_2_O_2_/H_2_SO_4_ solution to modify the electrolyte affinity. The characteristics of the composite separator and the pristine PE separator were evaluated in terms of morphology, microstructure, electrolyte wettability, thermal shrinkage, and ionic conductivity. Moreover, CR2032-type unit half-cells composed of a LiFePO_4_ cathode, a Li anode, and the separator were assembled. The cell performances were evaluated and compared.

## 2. Materials and Methods

### 2.1. Fabrication of the Composite Separator

**Figure 1:**
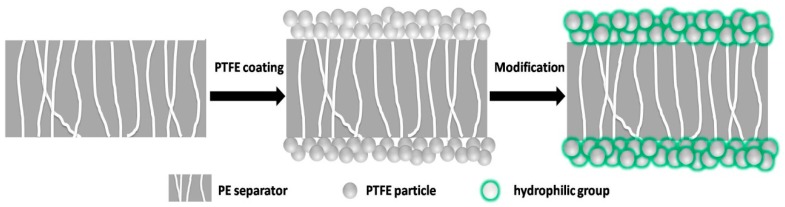
Schematic diagram for the fabrication of the composite separator. PTFE: polytetrafluoroethylene; PE: polyethylene.

A commercial PE separator (Tonen, thickness = 12 μm, F12BMS, Tokyo, Japan) was washed with acetone and dried at 50 °C for 12 h prior to use. A 60% content PTFE suspension from Sigma-Aldrich Company (Saint Louis, MO, USA) was adopted to coat the PE separators. Prior to the coating process, the suspension was diluted with deionized water to a solid content of 20 wt % under ultrasonic assistance. The fabrication of the composite separator was done by two main procedures following the scheme shown in Figure 1. First of all, the dilute suspension was applied onto both sides of the PE separators via a dip-coating process. The PTFE-coated separator was dried in a vacuum oven at 80 °C for 24 h. In order to enhance the electrolyte affinity of the PTFE coating layer, a milder chemical modification that avoided damaging the mechanical strength of the PTFE-coated separator was then adopted from Löhbach and Bakowsky [22]. The PTFE-coated separator was impregnated into a H_2_O_2_ (30%, Sinopharm Chemical Reagent Co., Ltd., Shanghai, China)/H_2_SO_4_ (98%, Sinopharm Chemical Reagent Co., Ltd., Shanghai, China) (1:1) solution and left at 50 °C for 30 s. Next, the separator was flushed with deionized water, followed by acetone, and then dried at 60 °C in a nitrogen atmosphere. The treated PTFE-coated separator was abbreviated as trPTFE-coated separator. The final thickness of the trPTFE-coated separator was measured to be around 18 μm.

### 2.2. Characterization of the Composite Separator

The surface morphology of the separators was examined using a scanning electron microscope (JEOL, JSM-6300, Akishima, Tokyo, Japan). The average pore size of the separators was evaluated using a mercury porosimeter (Micromeritics, Auto Pore IV9500, Norcross, GA, USA). The porosity of the coating layers was measured in accordance with the density method [23], calculated by the following equation:

(1) $$\mathrm{Porosity}=\frac{\rho_{c}-\rho_{e}}{\rho_{c}}$$

where ρ_c_ is the PTFE density for a dense coating layer without pores (theoretical value 2.20 g·cm^−3^) and ρ_e_ is the experimental density of a porous coating layer, which is equal to the mass difference of the PE separator before and after the coating divided by the measured volume of the PTFE coating. The trPTFE-coating was investigated to further verify the structure variation via FT-IR (JASCO, FT-IR 4100, Hachioji, Tokyo, Japan). The spectra were recorded at room temperature in the wave number range from 3500 to 500 cm^−1^. The electrolyte used in the work was 1 M LiPF_6_/EC + DEC (1/1, *v*/*v*). The electrolyte hydrophilicity of the separators was characterized by the contact angle measurement. The contact angle of the electrolyte on the separators was measured in air at room temperature using the sessile drop method with a contact angle meter (Chengde Jinhe Technic Apparatus Co. Ltd., JC2000C1, Chengde, China). The electrolyte uptake of the separators was calculated by the following equation:

(2) $$\mathrm{Uptake}=\frac{W-W_{o}}{W_{o}}$$

where W_o_ and W are the weights of the separator before and after soaking in the electrolyte. The ionic conductivity of the separators was measured with stainless steel (SS)/separators/SS blocking cell after being filled with the electrolyte by AC (alternating current) impedance measurement using an electrochemical workstation (Princeton Applied Research 273, Princeton, NJ, USA) at a frequency range from 100 kHz to 1 Hz with an amplitude of 10 mV. The electrochemical stability window of the separators was evaluated through linear sweep voltammeter (LSV) performed on a working electrode of SS and a counter and reference electrode of Li foil at a scan rate of 1.0 mV·s^−1^, with the potential ranging from 3 to 6 V. The differential scanning calorimeter (NETZSCH, STA 449F3, Schwanstetten, Bavaria, Germany) was used to determine the melting temperature of the separators. The thermal shrinkage of the separators as a function of time was determined by measuring the dimensional change at 130 °C. Photographs were taken of the separators after they were exposed to different temperatures for 0.5 h, and the photographs were examined. A puncture penetration test and a tensile strength test of the separators were conducted according to the method in [24] to investigate the separator’s resistance against the penetration of sharp objects such as Li dendrites. To evaluate the effects of the PTFE coating on cell performances, CR2032-type unit half-cells were assembled by sandwiching a separator between a Li foil anode and a LiFePO_4_ cathode and then activated with the electrolyte. All the assembly of the cells was carried out in an argon-filled glove box. The C-rate capability and cyclability of the cells were examined. The cells were cycled at several discharging rates varying from 0.5 C to 4 C (0.5 C, 1 C, 2 C, and 4 C) at a constant charging rate of 0.5 C under a voltage range from 2.5 to 4.2 V. The cells were cycled at a constant charge/discharge rate of 0.5 C/0.5 C for 100 cycles to evaluate the cycling performance.

## 3. Results and Discussion

**Figure 2:**
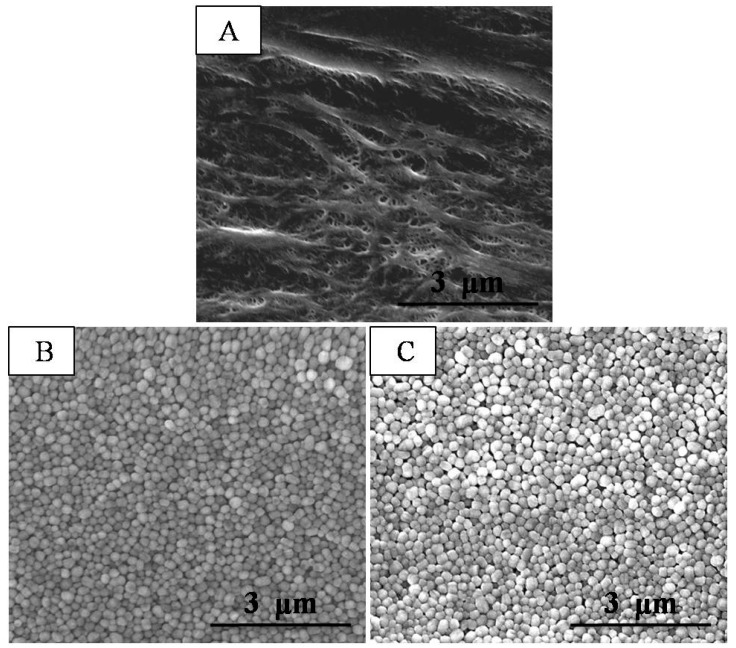
SEM images of (**A**) the PE separator; (**B**) the PTFE-coated separator and (**C**) the trPTFE-coated separator.

SEM images of the composite separators were compared with those of a commercial PE separator. Currently, a PE separator with a microporous structure is widely used in the Li-ion battery field; however, the pore size and distribution are not uniform enough. As shown in Figure 2A, the pristine PE separator showed a typical arborization porous structure after undergoing a wet process. In contrast to the pristine PE separator, the composite separators (Figure 2B,C) exhibited unique coating layers composed of close-packed PTFE particles (about 150 nm in diameter), which contributed to a well-developed porous structure. Comparing Figure 2B with Figure 2C, there is almost no visible change before and after the milder chemical modification, which is consistent with a previous study [20]. This indicates that the treatment did not significantly affect the porous structure of the coating layer. In order to provide quantified datum, the porosity of the coating layers and average pore size of the separators were examined (Table 1). Compared with the porosity of the PE separator, the porosity of the PTFE coating layers was observed to be about 65% due to the homogeneous alignment of the PTFE nanoparticles. The average pore size of the PTFE-coated separator was about 40 nm. The above results confirm that the PTFE coating layers allow for the development of a porous structure.

**Figure 3:**
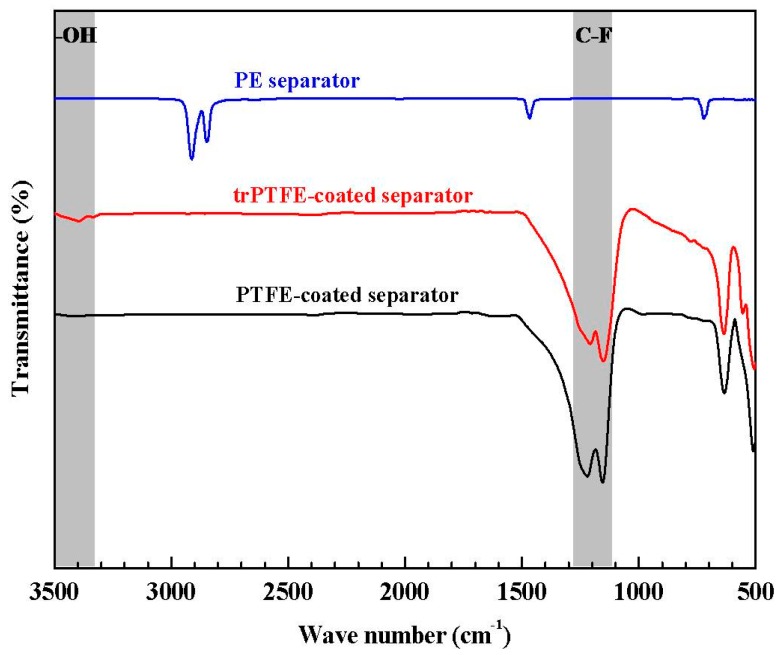
FT-IR spectra of the PE separator, the PTFE-coated separator, and the trPTFE-coated separator.

During the modification reaction, the H_2_O_2_/H_2_SO_4_ solution attacks the C–F bonds of PTFE, which induces a defluorinated reaction. Meanwhile, the bond formation between the introduced hydroxy groups and carbon occurs. In order to further verify the structural variation of the PTFE coating layer before and after the chemical modification, FT-IR measurements of the PTFE-coated separator and trPTFE-coated separator were obtained between 3500 and 500 cm^−1^, as shown in Figure 3. Due to the presence of the coating layer, the characteristic peak of PE completely disappears. It can be seen that the PTFE-coated separator shows the typical absorption bands of C–F bonds in the region of 1300 to 1100 cm^−1^. In addition to the C–F bands, the trPTFE-coated separator shows a new broad absorption band between 3500 and 3300 cm^−1^. Based on the previous study [20], this band belongs to the OH group, owing to the milder chemical modification. This change is expected to obviously enhance the electrolyte affinity of the trPTFE-coated separator.

**Figure 4:**
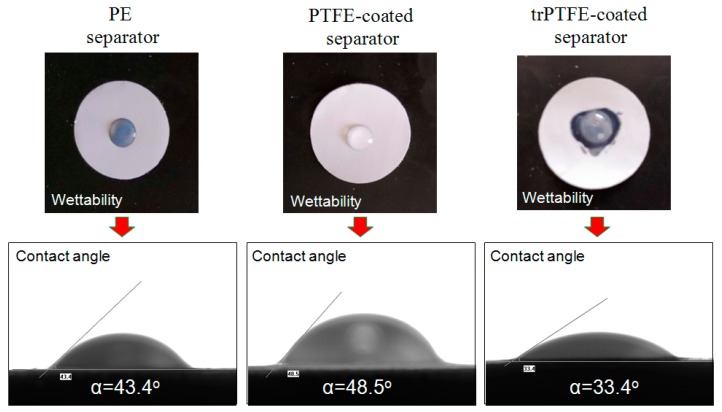
Liquid electrolyte wettability of the separators and the corresponding contact angle.

The wetting behavior of the separators was investigated using a liquid electrolyte absorption test. The corresponding static electrolyte contact angle is shown in Figure 4 and also listed in Table 1. As can be seen in Figure 4, the liquid droplet remained steady on the PE separator for a few minutes, and the PTFE-coated separator was hardly wetted by the liquid electrolyte due to the strong hydrophobicity of the unmodified PTFE layer. In contrast, the trPTFE-coated separator soaked up a portion of the electrolyte immediately, where the electrolyte droplet could easily spread over surrounding area. Because the separator pores were filled with the electrolyte, the trPTFE-coated separator became partly transparent. The static electrolyte contact angle test results were similar to the electrolyte absorption test. In comparison with the contact angle (43.4°) of the PE separator, that of the PTFE-coated separator was observed to be 48.5°, while the contact angle of the trPTFE-coated separator was significantly decreased to 33.4°. These results demonstrate that the improvement in the electrolyte wettability of the trPTFE-coated separator is remarkable, owing to the milder chemical modification. This result also verifies the change in the surface groups of the PTFE coating layer before and after the modification, as mentioned in Figure 3. The electrolyte uptake is a more straightforward parameter of wettability for separators used in batteries. The electrolyte uptake of the trPTFE-coated separator was more than 190%, which was clearly higher than that of the PE separator and that of the PTFE-coated separator (Table 1). It can be concluded that the trPTFE-coated separator exhibited outstanding electrolyte wettability, owing to its well-defined porous structure and its surface hydrophilicity, as verified above.

**Figure 5:**
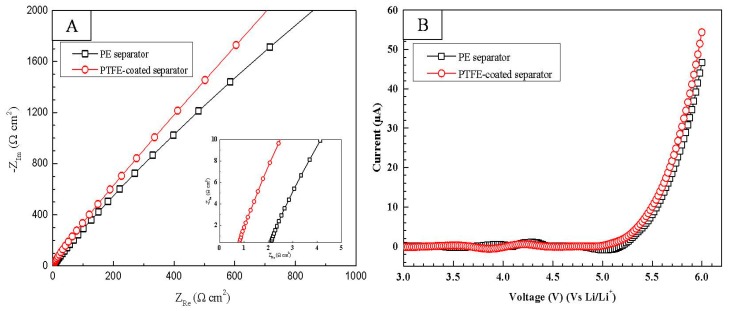
(**A**) Nyquist plots of the cells with the PE separator and the trPTFE-coated separator. The insert is the partial enlarged drawing; (**B**) The linear sweep voltammogram of the cells with the PE separator and the trPTFE-coated separator.

Figure 5A shows the Nyquist plots of the PE separator and the trPTFE-coated separator. As shown in Figure 5A, the impedance spectra show the intercepts of the inclined spike on the real axis, which represent the bulk resistance (R_b_) of the separators. The ionic conductivity was calculated based on the following equation:

(3) $$\sigma =\frac{t}{R_{b}A}$$

where, σ is the ionic conductivity, R_b_ is the bulk resistance, and t and A are the thickness and area of the separators, respectively. The ionic conductivity was calculated to be 2.5 × 10^−4^ S cm^−1^ for the PE separator, while the trPTFE-coated separator exhibited a higher ionic conductivity of 9.6 × 10^−4^ S cm^−1^. This result also shows that high porosity and good surface wettability contribute to the enhancement of the ionic conductivity. The electrochemical stability of the separators can be evaluated by linear sweep voltammogram (LSV). The LSV curves of the cells with the PE separator and the trPTFE-coated separator are shown in Figure 5B. It is apparent that both curves rose sharply until the voltage reached 5.0 V. This result implies that the electrochemical stability of the trPTFE-coated separator is comparable to that of the PE separator, and no decomposition of any components below 5.0 V (vs. Li/Li^+^) took place. Therefore, the trPTFE-coated separator can be used as a reliable alternative to the PE separator, even in application to high-voltage Li-ion batteries.

**Figure 6:**
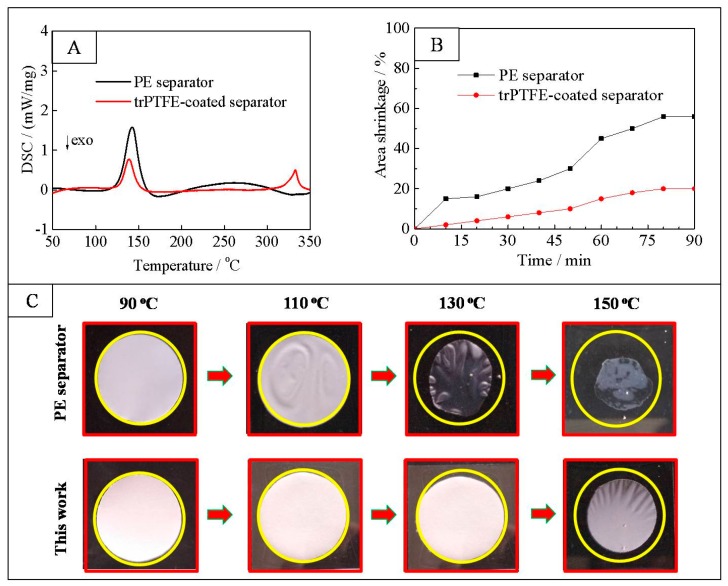
(**A**) DSC curves of the PE separator and the trPTFE-coated separator; (**B**) Thermal shrinkage of the separators as a function of time at 130 °C; and (**C**) Photographs of the separators after being exposed to 90, 110, 130, and 150 °C for 0.5 h, respectively.

Figure 6A shows the DSC curves of the PE separator and the trPTFE-coated separator. As displayed in the DSC curve of the PE separator, an endothermic peak occurring at 142.7 °C can be observed, which is in accordance with the melting point of PE. For the trPTFE-coated separator, two endothermic peaks appearing at 140.5 and 332.7 °C belong to the melting points of PE and PTFE, respectively. The DSC results indicate that the trPTFE-coated separator displayed a higher shutdown temperature than the PE separator because of the presence of the PTFE resin. Figure 6B shows the evolution of the thermal shrinkage of the separators as a function of time at 130 °C. The thermal shrinkage of both separators increases as a function of time. In comparison with the PE separator, the trPTFE-coated separator exhibited a lower area shrinkage at the same temperature. Up to 90 min, the shrinkage of the trPTFE-coated separator was still under 20%, which was far lower than that of the PE separator (55%). Figure 6C depicts the photographs of the separators after being exposed at 90, 110, 130, and 150 °C for 0.5 h, respectively. Both separators remained stable under 100 °C. The PE separator lost dimensional stability upon exposure to high temperatures of 130 °C, while the trPTFE-coated separator suppressed the thermal shrinkage compared with the PE separator under the same conditions. Because the PE resin has a melting point of about 140 °C, as proven above, the PE separator tends to melt when exposed to temperatures of 150 °C. Fortunately, the trPTFE-coated separator can maintain its original shape to some degree, owing to the high-melting point of the PTFE coating layer. As with other ceramic particles, the heat resistant PTFE particles are believed to effectively prevent the composite separator from being thermally shrunk. So, the excellent thermal stability of the trPTFE-coated separator could effectively prevent an internal electrical short circuit at elevated temperatures.

The mechanical strength of the separator plays a critical role in suppressing Li dendrites. The puncture resistance and tensile strength of the pristine PE separators, PTFE-coated separator, and trPTFE-coated separator are shown in Table 1. According to the results, the puncture resistance of the trPTFE-coated separator was improved by 14% compared with that of the PE separator, and the tensile strength of the trPTFE-coated separator was a little higher than that of the PE separator. The improvement of the mechanical strength was mainly due to the formation of a uniform PTFE coating on the surface of the PE separator.

**Figure 7:**
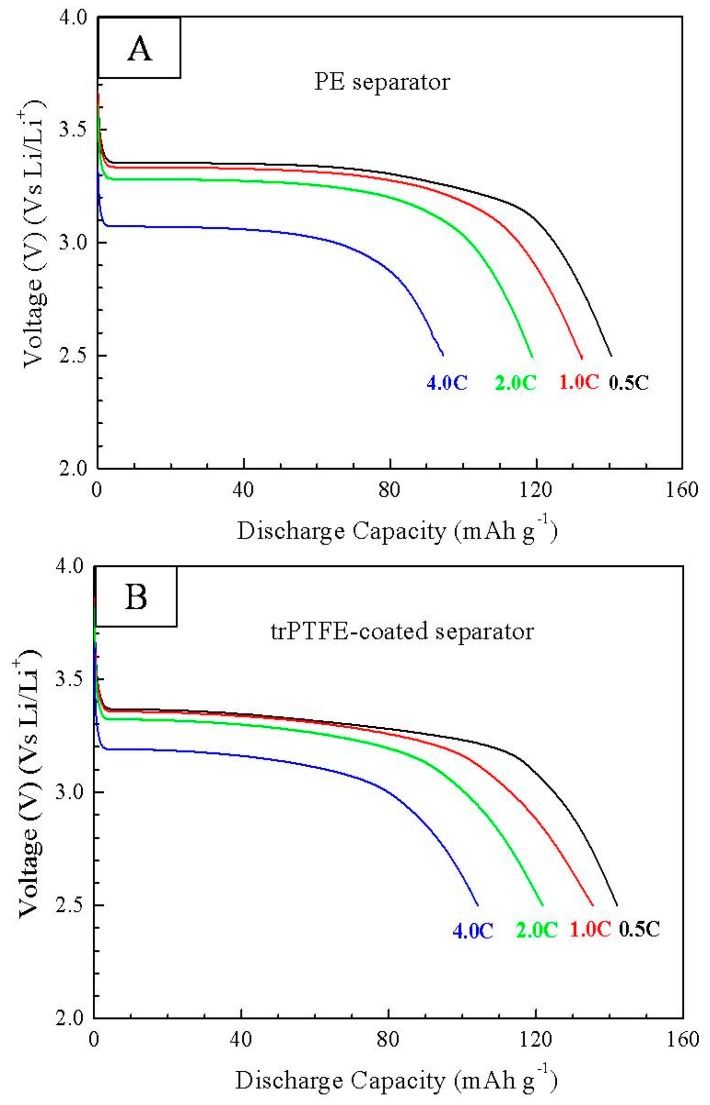
C-rate capability of the cells with (**A**) the PE separator and (**B**) the trPTFE-coated separator at 0.5 C, 1 C, 2 C, and 4 C.

Figure 7 shows the C-rate capability of the cells with the PE separator and the trPTFE-coated separator. The discharge capacity of both cells gradually decreased with an increase of the discharge current density. No unstable discharge phenomenon was observed for the trPTFE-coated separator. Moreover, the discharge capacity of the trPTFE-coated separator (141.9 mAh/g at 0.5 C) appeared to be a little higher than that of the PE separator (140.3 mAh/g at 0.5 C). When the discharge current was increased to a 4.0 C rate, the advantage of the trPTFE-coated separator in the discharge C-rate capacity became more pronounced. The discharge C-rate capacity of the trPTFE-coated separator was 104.2 mAh/g at 4.0 C, which was much higher than that of the PE separator (94.5 mAh/g at 4.0 C). These results demonstrate that the cell assembled with exhibited better C-rate capability than that of the PE separator, which reveals that the trPTFE-coated separator’s higher ionic conductivity due to its porosity and wettability could be beneficial in improving its discharge C-rate capability.

**Figure 8:**
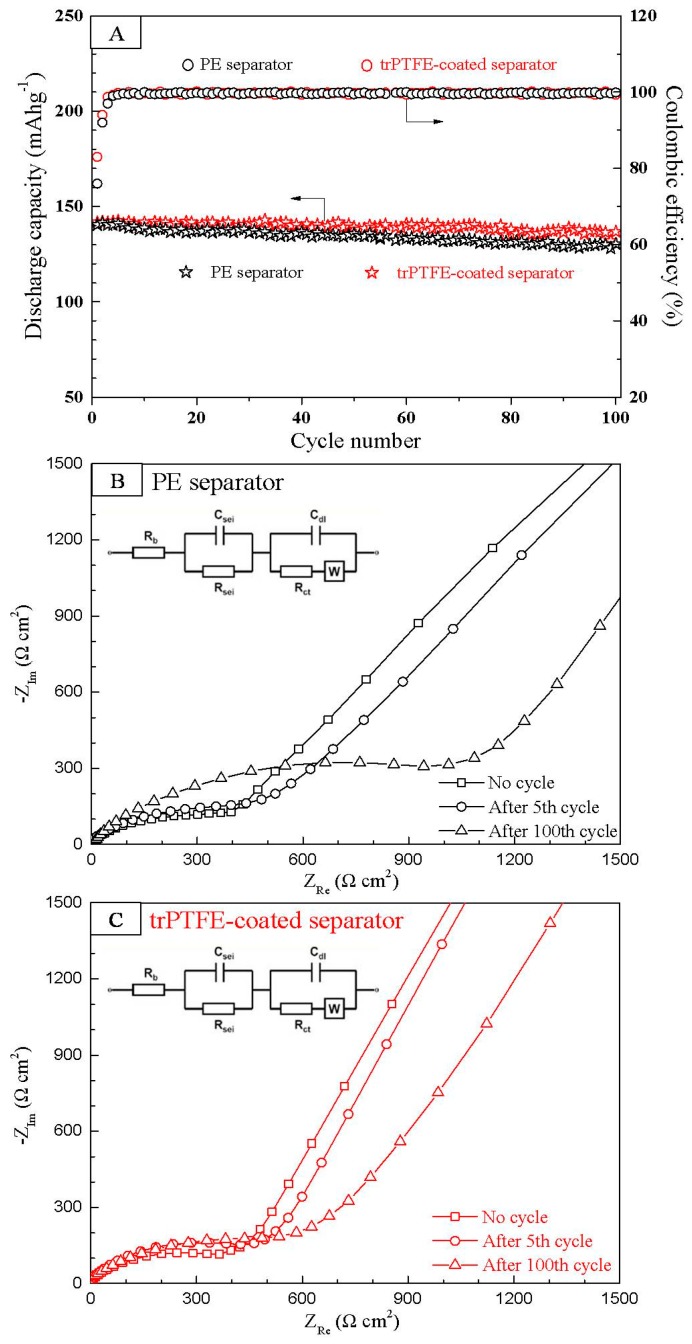
(**A**) The cyclability of the cells with the PE separator and the trPTFE-coated separator as a function of the cycle number (0.5 C/0.5 C); the impedance spectra of the cells with (**B**) the PE separator and (**C**) the trPTFE-coated separator after the 5th and 100th cycle. The inset is the equivalent circuit.

Figure 8A depicts the cyclability of the cells with the PE separator and the trPTFE-coated separator as a function of the cycle number. Both separators exhibited relatively stable coulombic efficiency. For the PE separator, its coulombic efficiency was only 76.1% when a current density of 0.5 C was used for both the charge and discharge processes, and then, its coulombic efficiency steeply increased to about 99.6% in the subsequent cycles. The trPTFE-coated separator showed a higher first coulombic efficiency (83.5%), though it had a similar tendency with respect to its coulombic efficiency, which may be attributed to its favorable electrolyte affinity. The discharge capacity of both cells decreased slightly with cycling, which may be ascribed to little change in internal impedance [1,25], and the discharge capacity of the trPTFE-coated separator appeared to be slightly higher than that of the PE separator up to 100 cycles. The capacity retention after the 100th cycle was found to be 97% for the trPTFE-coated separator and 91% for the PE separator, respectively. The excellent cyclability can be explained by previous studies [26,27]. The trPTFE-coated separator had superior wettability than the hydrophobic PE separator alone, which contributed to sufficient contact with the electrolyte during cycling. Moreover, the electrochemical impedance spectra of the cells after the 5th and 100th cycle were analyzed to evaluate the cyclability in this study, as shown in Figure 8B,C. The EIS (electrochemical impedance spectroscopy) data can be fitted by an equivalent circuit shown in the inset of Figure 8B,C. The R_b_ is the bulk resistance of the cell, which reflects the electric conductivity of the electrolyte, separator, and electrodes; R_sei_ and C_sei_ are the resistance and capacitance of the solid-state interface layer formed on the surface of the electrodes, respectively; R_ct_ and C_dl_ are the charge-transfer resistance and its relative double-layer capacitance, respectively; and W is the Warburg impedance related to a combination of the diffusional effects of the lithium ion on the interface. The main parameters obtained by fitting the Nyquist plots are listed in Table 2. The impedance of the cell assembled with the PE separator significantly increased after the 100th cycle, which had a negative influence on cell capacity. In comparison with the PE separator, the trPTFE-coated separator only increased a little in the cell impedance. This suppressed growth in the cell impedance may be attributed to its high electrolyte uptake in the cell during cycling, which confirms again the above conclusion.

**Figure 9:**
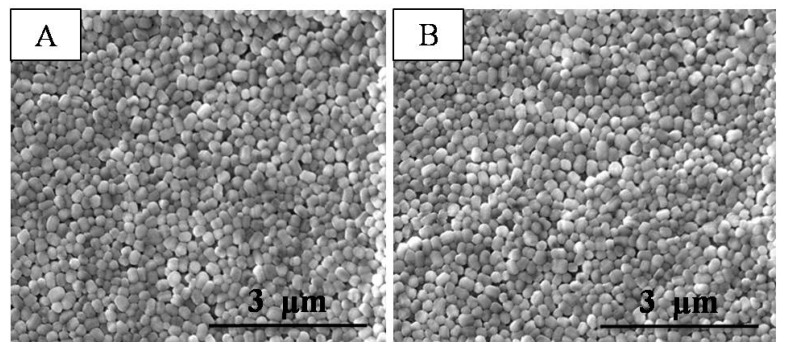
SEM of the trPTFE-coated separator after the 100th cycle: (**A**) cathode side; and (**B**) anode side.

In order to investigate the morphological stability after cycling, the cell with the trPTFE-coated separator was opened warily. SEM images of the trPTFE-coated separator after the 100th cycle are shown in Figure 9. Both sides of the trPTFE-coated separator had no obvious change. Neither breakdown of the porous structure nor peel-off of the PTFE particles was observed. All the positive results reveal that the PTFE coating layer was stable and hardly dissolved in the cell under the cycling condition. 

## 4. Conclusions

A novel self-binding PTFE-coated separator was successfully prepared by coating PTFE particles on both sides of a porous PE separator using a dilute PTFE suspension. Then, the hydrophobicity of the composite separator was modified by a H_2_O_2_/H_2_SO_4_ solution, which was attributed with the introduction of OH groups on the surface of the coating layer. The coating layer, consisting of close-packed PTFE particles, possessed a highly ordered nano-porous structure (trPTFE coating porosity 66%) and excellent electrolyte uptake (190.6%), which resulted in considerable ionic conductivity (almost four times higher than that of the PE separator). Due to the presence of the high-melting point PTFE particles (332.7 °C), the composite separator exhibited good thermal stability. Li-ion cells composed of a LiFePO_4_ cathode, a Li anode, and the composite separator were assembled. The discharge capacity of the trPTFE-coated separator appeared to be higher than that of the PE separator, especially at higher discharge currents. The discharge C-rate capacity of the trPTFE-coated separator was 104.2 mAh/g at 4.0 C, which was much higher than that of the PE separator (94.5 mAh/g at 4.0 C). Owing to sufficient contact between the electrolyte and the composite separator during cycling, the cell showed good cyclability. After 100 cycles, the PTFE coating layer was still stable and hardly dissolved in the cell.

## Figures and Tables

**Table 1:** Basic characteristics of the separators: porosity, contact angle, and electrolyte uptake.

	PE Separator	PTFE-Coated Separator	trPTFE-Coated Separator
Thickness (μm)	12	18	18
Porosity (coating layer/PE separator) (%/%)	-/45	65/45	66/45
Average pore size (nm)	43	40	40
Contact angle (°)	43.4	48.5	33.4
Electrolyte uptake (%)	110.7	171.5	190.6
Puncture resistance (gf)	320.5	368.6	365.8
Tensile strength (kgf)	3.2	3.6	3.6

**Table 2:** The main parameters obtained by fitting the Nyquist plots.

	R_b_ (Ω)	R_sei_ (Ω)	R_ct_ (Ω)
PE separator-No cycle	2.5	93.1	341.5
PE separator-5th cycle	3.1	101.4	370.2
PE separator-100th cycle	5.4	280.5	733.2
trPTFE-coated separator-No cycle	1.1	80.6	290.3
trPTFE-coated separator-5th cycle	1.5	85.2	304.2
trPTFE-coated separator-100th cycle	4.1	166.5	441.7
